# Causal association of lipoprotein-associated phospholipids on the risk of sepsis: a Mendelian randomization study

**DOI:** 10.3389/fendo.2023.1275132

**Published:** 2024-01-11

**Authors:** Liying Zeng, Haoxuan Tang, Jiehai Chen, Yijian Deng, Yunfeng Zhao, Hang Lei, Yufei Wan, Ying Pan, Yongqiang Deng

**Affiliations:** ^1^ Guangdong Provincial Key Laboratory of Proteomics, State Key Laboratory of Organ Failure Research, Department of Pathophysiology, School of Basic Medical Sciences, Southern Medical University, Guangzhou, China; ^2^ Department of Anesthesiology, Nanfang Hospital, Southern Medical University, Guangdong Provincial Key Laboratory of Precision Anaesthesia and Perioperative Organ Protection, Guangzhou, Guangdong, China; ^3^ School of Traditional Chinese Medicine, Southern Medical University, Guangzhou, Guangdong, China

**Keywords:** lipoprotein-associated phospholipids, sepsis, Mendelian randomization, causal relationship, genetics

## Abstract

**Background:**

Many previous studies have revealed a close relationship between lipoprotein metabolism and sepsis, but their causal relationship has, until now, remained unclear. Therefore, we performed a two-sample Mendelian randomization analysis to estimate the causal relationship of lipoprotein-associated phospholipids with the risk of sepsis.

**Materials and methods:**

A two-sample Mendelian randomization (MR) analysis was performed to investigate the causal relationship between lipoprotein-associated phospholipids and sepsis based on large-scale genome-wide association study (GWAS) summary statistics. MR analysis was performed using a variety of methods, including inverse variance weighted as the primary method, MR Egger, weighted median, simple mode, and weighted mode as complementary methods. Further sensitivity analyses were used to test the robustness of the data.

**Results:**

After Bonferroni correction, the results of the MR analysis showed that phospholipids in medium high-density lipoprotein (HDL; OR_IVW_ = 0.82, 95% CI 0.71-0.95, *P* = 0.0075), large HDL (OR_IVW_ = 0.92, 95% CI 0.85-0.98, *P* = 0.0148), and very large HDL (OR_MR Egger_ = 0.83, 95% CI 0.72-0.95, *P* = 0.0134) had suggestive causal relationship associations with sepsis. Sensitivity testing confirmed the accuracy of these findings. There was no clear association between other lipoprotein-associated phospholipids and sepsis risk.

**Conclusions:**

Our MR analysis data suggestively showed a correlation between higher levels of HDL-associated phospholipids and reduced risk of sepsis. Further studies are required to determine the underlying mechanisms behind this relationship.

## Introduction

In 2016, the Third International Consensus Definition for Sepsis and Septic Shock (Sepsis-3) defined sepsis as a life-threatening organ dysfunction resulting from dysregulated host responses to infection, and emphasized the primacy of the non-homeostatic host response to infection (1). Sepsis is a life -threatening disease with a high incidence and it remains one of the main causes of death globally. In 2017, an estimated 48.9 million incident cases of sepsis and 11 million sepsis-related deaths were reported worldwide, representing 19.7% of all global deaths (2). Sepsis is therefore significant public health problem with considerable economic consequences.

Based on differences in density, size, lipid and apolipoprotein composition, lipoproteins can be divided into five main subcategories: chylomicrons, very low-density lipoprotein (VLDL), low-density lipoprotein (LDL), intermediate-density lipoprotein (IDL) and high-density lipoprotein (HDL) (3). The lipids found in HDL are mainly surface phospholipids, internal cholesterol esters, and triglycerides. Phospholipids are mainly phosphatidylcholine, lysophosphatidylcholine, and sphingomyelin, each accounting for 32 – 35 mol%, 1.4 – 8.1 mol%, and 5.6 – 6.6 mol% of total lipids in HDL, respectively (4). At present, various activities of HDL have been reported, mainly including anti-inflammatory, anti-oxidative, immunomodulatory, anti-apoptotic, endothelial, and anti-thrombotic functions (5–8). Studies have shown that serum and HDL phospholipids are significantly reduced after a single intravenous dose of endotoxin (9). Phospholipids have also been suggested to play a specific role in HDL’s protective capacity against the pro-inflammatory effects of C-reactive protein (CRP). A study has shown that HDL blocks the upregulation of CRP-induced inflammatory adhesion molecules through its phospholipid components (10). In addition, when recombinant HDL made with HDL apolipoprotein and phosphatidylcholine (PC) instead of saline was administered intravenously 3.5 hours before a single intravenous dose of endotoxin, healthy volunteers showed fewer flu-like symptoms and lower plasma inflammatory cytokines (11). These data suggest that the anti-inflammatory and immunomodulatory effects of HDL are closely related to its phospholipids. Although Mendelian randomization (MR) analysis has shown a causal relationship between high levels of high-density lipoprotein cholesterol and a reduced risk of infectious hospitalizations (12). However, the causal relationship between HDL-related phospholipids and infectious diseases or sepsis is unclear.

One observational study identified a reduction in LDL, HDL, and HDL-associated apolipoproteins in non-survivors of sepsis compared with survivors (13). LDL can reduce sepsis-associated lipopolysaccharide (LPS) damage by binding to LPS (14). In mouse models, the lethal effects of LPS were prevented by removing LDL receptor-induced high endogenous LDL levels (15). Thus, LDL can partially reduce the degree of LPS-induced post-inflammatory acute phase response. Other studies have shown that triglyceride-rich lipoproteins, such as chylomicrons and VLDL, also can inactivate LPS and prevent endotoxin-induced rodent deaths (16–18). IDL is a residual lipoprotein produced by VLDL hydrolysis. In a large study of more than 7,000 participants, endotoxemia was found to be associated with high concentrations of VLDL, IDL, and LDL particles, as well as low concentrations of HDL particles (19). Although the surface of all lipoproteins is composed of phospholipids, mainly PC and sphingomyelins (SM), there are significant differences in phospholipid species for different lipoproteins. Therefore, various lipoproteins and their phospholipids have different functions, and this study aims to explore the causal relationship between lipoprotein- associated phospholipids and sepsis.

Mendelian randomization (MR) refers to a statistical method based on genome-wide association studies (GWAS) that use genetic variation as instrumental variables (IVs) to assess the causality of observed associations between modifiable exposures or risk factors and clinically relevant outcomes (20). MR minimizes traditional confounding and reverses causation because genetic variants are randomly distributed during meiosis and are independent of environment, disease onset, and progression (21). Therefore, MR is not affected by the confounding biases found in traditional observational studies. Based on this knowledge, we applied a two-sample MR analysis to comprehensively investigate the genetic association of lipoprotein-associated phospholipids with sepsis. The results of this study may offer novel strategies for personalized treatments for sepsis.

## Materials and methods

### MR analysis

**Figure 1:**
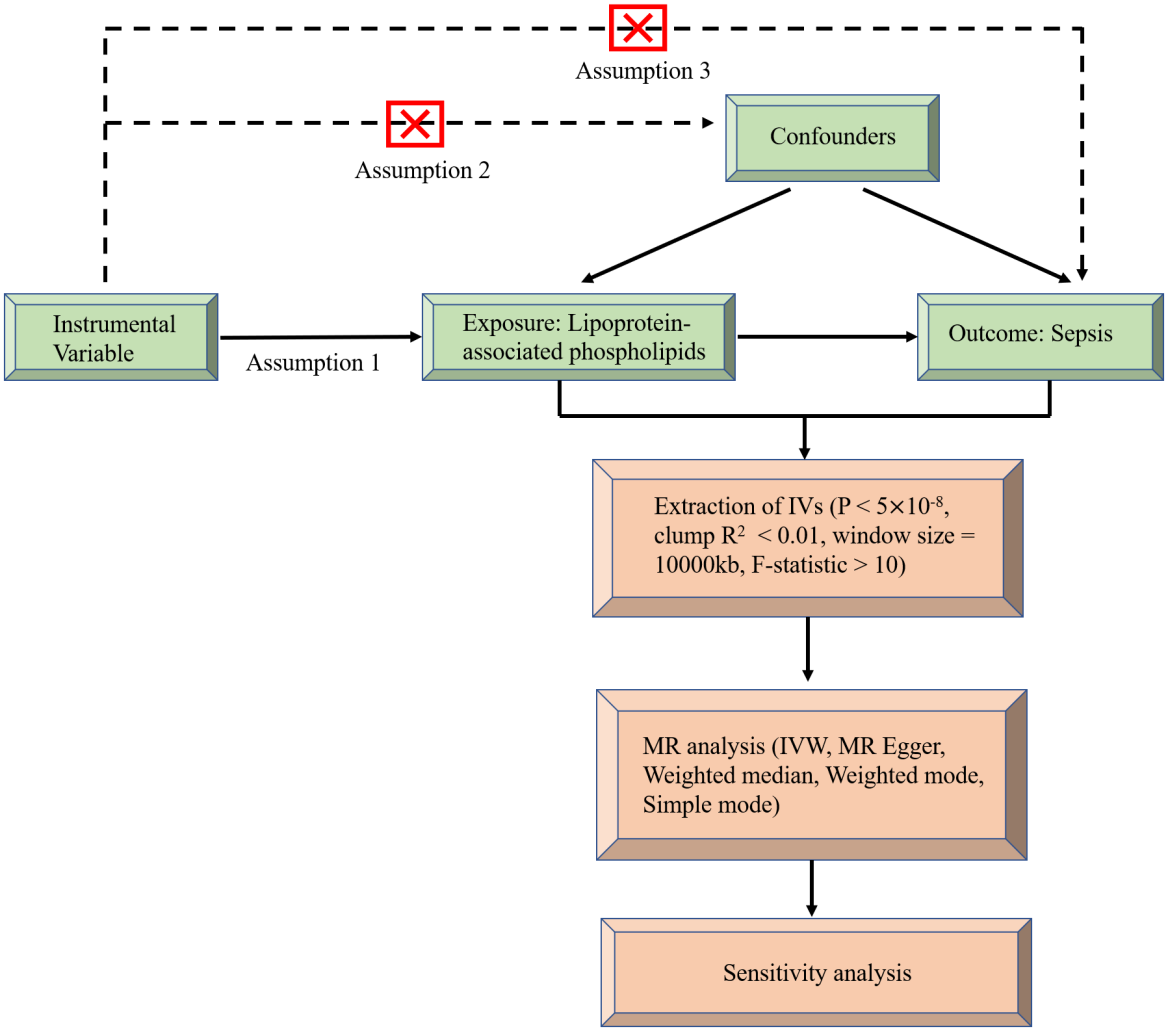
Flow chart of causal inference between lipoprotein-associated phospholipids and sepsis.

MR uses genetic variation as a tool variable to assess non-confounding causal relationships between exposure and outcome and it must satisfy the following three assumptions (22): (1) There is a strong correlation between instrumental variables and exposure factors, (2) there is no connection between instrumental variables and confounding factors, and they are independent of each other, (3) the instrumental variables are associated with outcomes only by exposure, and there is no direct correlation. A flowchart of causal reasoning for lipoprotein-associated phospholipids and sepsis is depicted in **Figure 1**. In short, lipoprotein-associated phospholipids were classed as the exposure and sepsis was the result. Single nucleotide polymorphisms (SNPs) significantly associated with lipoprotein-associated phospholipids were selected as IVs based on strict inclusion and exclusion criteria. A series of heterogeneity and sensitivity analyses were performed to identify significant associations.

### Data sources

This study used publicly available databases. Up to 24,925 individuals were tested in 14 genotype datasets from 10 European studies using additive genetic models. A genome-wide single nucleotide polymorphism (SNP) panel of 39 million genetic markers was tested for univariate associations with the concentrations of lipids and metabolites in 123 humans quantified by high-throughput NMR spectroscopy metabolomics. Individual lipoprotein phospholipids were analyzed using a high-throughput serum Nuclear Magnetic Resonance (NMR) metabolomics platform. The method provided serum measurement information, including lipoprotein subclass distribution and lipoprotein particle concentration, as well as detailed molecular information of serum lipids, including free and esterified cholesterol, sphingomyelin, and fatty acid saturation. (23). Individuals on lipid-lowering medications or pregnant individuals were excluded from the analyses. Genetic statistics for sepsis were derived from the UK Biobank, and we identified 11,643 cases of sepsis, with 474,841 controls of European ancestry. All cases were adjusted for age, sex, chip, and the first 10 principal component analysis.

### Selection of IVs

We extracted qualified IVs according to strict selection criteria. First, we selected independent SNPs closely related to various lipoprotein-associated phospholipids and *P* < 5×10^-8^ as potential IVs, respectively. Second, the European-based 1,000 Genome Projects reference panel was used to calculate the linkage disequilibrium (LD) and the threshold for clumping was set to R^2^ < 0.01, while the clumping window size was set to 10,000 kb. Third, SNPs with palindromic and a minor allele frequency (MAF) of less than 0.3 were also eliminated. Finally, IV strength was assessed using the F-statistic to further extract SNPs that were closely related to the exposure. If F > 10, the results should not suffer from weak instrument bias (24).

### Statistical analysis

R version 4.3.0 (R Foundation for Statistical Computing, Vienna, Austria) and the Two-Sample MR package were used for statistical analyses (25). *P* values < 0.05 were to be statistically significant. We performed MR analysis in five different ways: inverse variance weighted (IVW) as the primary method, MR Egger, weighted median, simple mode, and weighted mode as complementary methods (26–28).

We used Cochran’s Q statistic to detect heterogeneity in MR analyses, with *P* values > 0.05 indicating no heterogeneity (25). If heterogeneity was present, the random-effects model of the IVW method was used. If heterogeneity was absent, then a random-effects analysis was equivalent to a fixed-effect analysis. (29, 30). MR Egger regression was used to examine the effect of horizontal pleiotropy, and *P* values > 0.05 were indicative of no horizontal pleiotropy (31). Therefore, in the absence of pleiotropy, the IVW analysis method was preferred, and in the presence of pleiotropy, the MR Egger regression method was used (32). MR Egger regression can detect pleiotropy. To test the effect of each SNP on the results, we used leave-one-out analysis to determine whether the estimates were biased or driven by outliers (30). We corrected multiple comparisons using the Bonferroni method and set the statistical significance to P < 0.0042 (0.05/12) based on the number of exposures. If the P value was between 0.0042 and 0.05, we considered suggestive evidence of the potential causal associations (33).

## Results

Using the above method, SNPs were screened for this study and details of the selected SNPs are shown in **Table S1**. Further, the causal effects of each SNP on sepsis are shown in the forest plot (**Figure S1**).

**Figure 2:**
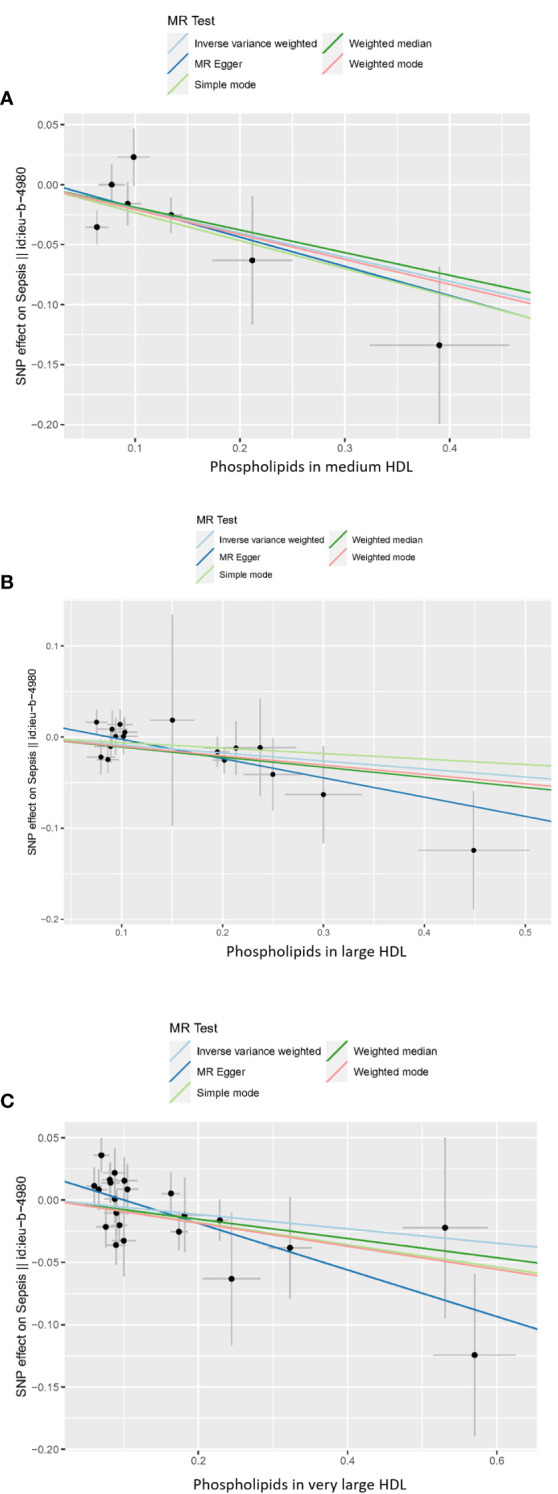
Scatter plot of the causal relationship between HDL-associated phospholipids and sepsis. **(A)** Phospholipids in medium HDL. **(B)** Phospholipids in large HDL. **(C)** Phospholipids in very large HDL. Analyses were conducted using IVW, weighted median, weighted mode, simple mode, and MR Egger methods. The slope of the line indicates the magnitude of the causal relationship. Error bars indicate 95% CI. MR, mendelian randomization; SNP, single nucleotide polymorphism.

**Figure 3:**
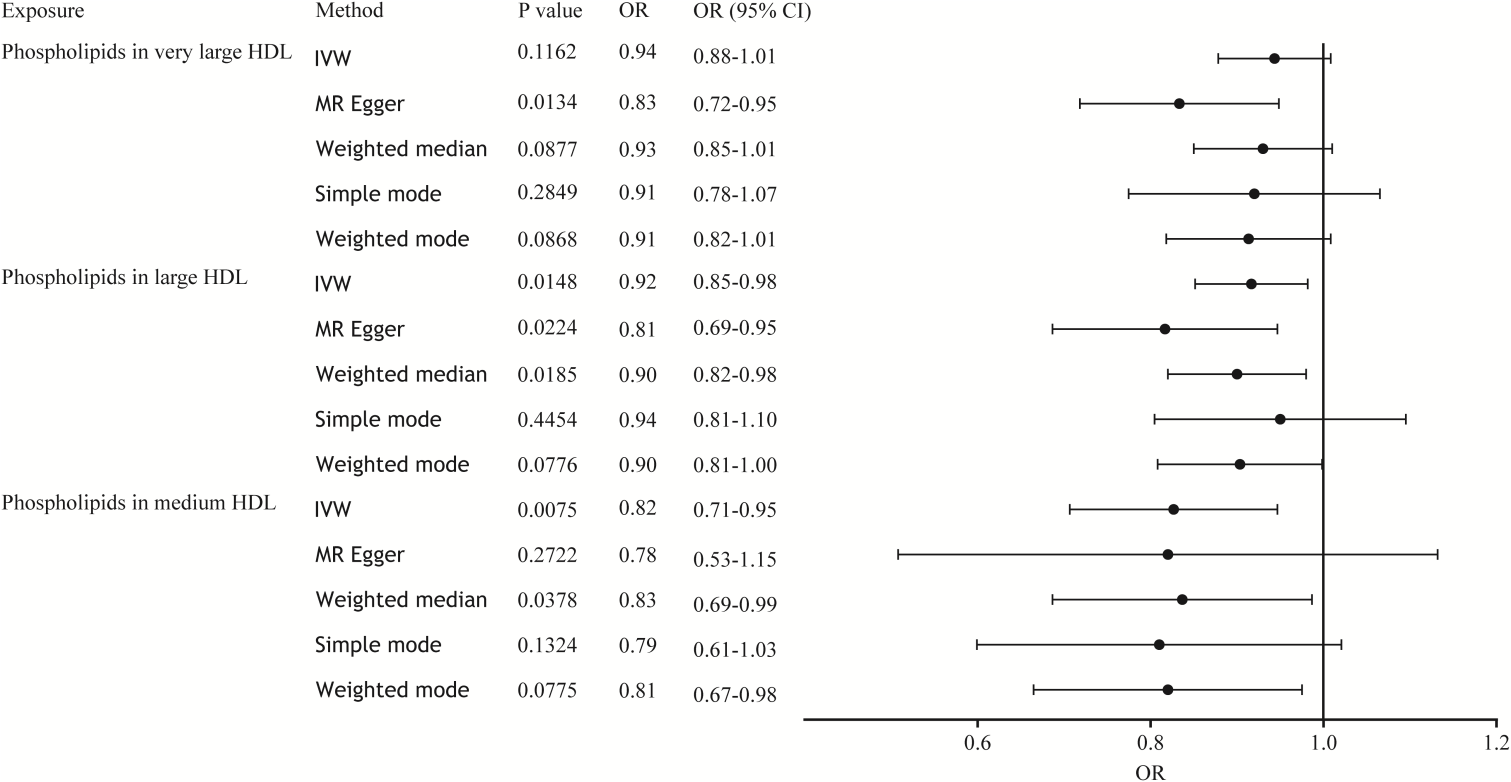
Forest plot of the causal relationship between HDL-associated phospholipids and sepsis. The black dots represent the OR value obtained by each method and the solid line represents the 95% CI. IVW, inverse variance weighted; MR, Mendelian randomization; OR, odds ratio; CI, confidence interval.

In the MR analysis, we used a variety of methods to assess the causal relationship between lipoprotein-associated phospholipids and sepsis. The results of the MR analysis suggestively showed that HDL-associated phospholipids are causally related to sepsis (**Figure 2**). Phospholipids in medium HDL (OR_IVW_ = 0.82, 95% CI 0.71-0.95, *P* = 0.0075) and phospholipids in large HDL (OR_IVW_ = 0.92, 95% CI 0.85-0.98, *P* = 0.0148) were negatively associated with sepsis. Due to the pleiotropy of phospholipids in very large HDL, the MR Egger regression method was used (OR_MR Egger_ = 0.83, 95% CI 0.72-0.95, *P* = 0.0134). However, phospholipids in LDL, IDL, VLDL, and chylomicrons were not causally associated with sepsis (**Figure S2**; **Table S2**). Thus, only three HDL-associated phospholipids (phospholipids in medium HDL, phospholipids in large HDL, and phospholipids in very large HDL) suggestively showed significant causal relationship associations with sepsis (**Figure 3**).

In sensitivity analysis, we performed heterogeneity, pleiotropy, and leave-one-out analysis to assess the reliability and robustness of the results (**Table 1**). Cochran’s Q test showed no heterogeneity between IVs (phospholipids in medium HDL, *P*_IVW_ = 0.277, *P*_MR Egger_ = 0.1914; phospholipids in large HDL, *P*_IVW_ = 0.7786, *P*_MR Egger_ = 0.8902; phospholipids in very large HDL, *P*_IVW_ = 0.1084, *P*_MR Egger_ = 0.2545). The symmetry of the funnel plot also confirmed the absence of heterogeneity, suggesting that causal associations were less likely to be affected by potential bias (**Figure S3**). Therefore, the random-effects model of the IVW method was used. To reduce bias due to horizontal pleiotropy, we performed MR Egger intercept testing, which showed that overall horizontal pleiotropism was absent in all IVs (phospholipids in medium HDL, *P* = 0.8224; phospholipids in large HDL, *P* = 0.1190). When horizontal pleiotropy is present, the MR Egger regression method can be used in MR analysis (phospholipids in very large HDL, *P* = 0.0435). The leave-one-out analysis (**Figure S4**) showed no substantial difference in the estimated causal effect when individual SNPs were systematically removed and the MR analysis was repeated. This showed a strong association between exposure and outcomes, thereby validating the reliability of the results of this study.

**Table 1:** Heterogeneity and pleiotropy analysis of MR.

Exposure	Analysis	Methods	Effect size	*P* value
Phospholipids in medium HDL	Heterogeneity	Cochran’s Q test	7.501 (Q_IVW_)	0.277
			7.418(Q_MR Egger_)	0.1914
	Pleiotropy	MR Egger regression	0.0051 (egger_intercept)	0.8224
Phospholipids in large HDL	Heterogeneity	Cochran’s Q test	11.4849 (Q_IVW_)	0.7786
			8.7506 (Q_MR Egger_)	0.8902
	Pleiotropy	MR Egger regression	0.0186 (egger_intercept)	0.119
Phospholipids in very large HDL	Heterogeneity	Cochran’s Q test	29.2373 (Q_IVW_)	0.1084
			23.7268(Q_MR Egger_)	0.2545
	Pleiotropy	MR Egger regression	0.0188(egger_intercept)	0.0435

## Discussion

The inference of causality in genetics was usually made by MR analysis. MR analysis was a form of instrumental variable analysis in which genetic marker SNPs were often used as tools to infer the causal effects of exposure variables on outcome variables. SNPs were the basis of genetic polymorphisms and lead to most of the genetic differences between individuals. It was well known that SNPs had a close relationship with disease, and this relationship was the basis for understanding the etiology, medical prevention, and diagnosis of disease. Studies had found that various genetic and environmental factors could lead to abnormalities in blood lipids and lipoproteins, and it could be seen that plasma lipid and lipoprotein concentrations were highly heritable. In addition, in the field of lipid and lipoprotein metabolism, almost 30 different genes encoding key proteins had been implicated, and more than 200 different SNPs had been identified in these genes (34). The genetic variability involved could affect the final plasma lipid levels. The two-sample MR analysis used in this study showed that HDL-associated phospholipids had a suggestive causal relationship with sepsis. There were no clear associations between other lipoprotein-associated phospholipids and sepsis risk, including LDL, IDL, VLDL-associated phospholipids, and phospholipids in chylomicrons.

Sepsis remains the leading cause of morbidity and mortality worldwide, and its pathogenesis is complex and involves multiple interactions between infecting microorganisms and the host. Studies have found a rapid and significant decrease in serum cholesterol, phospholipids, apoB, and apoA-I carried in LDL and HDL during acute phase reactions to endotoxemia and various inflammatory in humans (35, 36). Among them, HDL phospholipids have been shown to selectively decrease, while the number of HDL particles remains unchanged. Phospholipids showed a greater decline of approximately 20%, and a highly statistically significant linear relationship between the percentage reduction in phospholipids and the peak CRP (R^2 =^ 0.97, *P* = 0.001) (9). Although all lipoproteins bind endotoxins, HDL is the most protective because it is rich in surface phospholipids (37). HDL is the main carrier of phospholipids in lipoproteins and has significant endotoxin neutralization ability. Infusion of HDL prevented the fatal consequences of LPS administration in mice (38) and prevented LPS-induced cytokine production in rabbits (39) and human volunteers (40). Sphingosine 1-phosphate (S1P) is a lipid-signaling molecule and approximately 55% and 35% of plasma S1P is partitioned into HDL and albumin, respectively (41). As an extracellular and intracellular messenger, S1P regulates the pathophysiological processes involved in sepsis progression. In patients with sepsis, serum S1P levels were significantly reduced, leading to sepsis capillary leakage, impaired tissue perfusion, and organ failure, which are all inversely correlated with disease severity (42). Other HDL-associated sphingolipids, such as sphingosinephosphorylcholine and lysosulfatide may also enhance endothelial cell migration, survival, and the cytoprotective effects of HDL (43). It could be seen that the serum HDL-associated sphingolipids level can predict the prognosis of sepsis, which provides ideas for the clinical treatment of sepsis patients.

One study found that phospholipid transfer active protein (PLTP) and endothelial lipase (EL) were significantly higher than in patients with non-sepsis (44). This lipase plays a major role in hydrolyzing the phospholipids in HDL (45). Studies have also shown that increased PLTP activity might promote phospholipid transfer from HDL to tissues (46). Increased transfer of HDL phospholipids to tissues might contribute to the regeneration of damaged cell membranes and lung surfactants, and endotoxins bound to HDL phospholipids might be excreted into the bile (47). Thus, increased PLTP activity during inflammation may be a protective mechanism that can attenuate the LPS response by modulating HDL phospholipids. This study revealed the potential causal role of HDL-associated phospholipids in sepsis, suggesting that therapeutic strategies to increase serum levels of HDL-associated phospholipids might be beneficial in patients with sepsis. However, further studies are required to improve our understanding of the mechanisms by which HDL-associated phospholipids affect various aspects of sepsis pathology.

Our MR analysis showed a correlation between elevated HDL-associated phospholipid levels and a reduced risk of sepsis. HDL was the main carrier of phospholipids in lipoproteins and had a significant endotoxin neutralizing ability. The core functions of HDL were considered to be antioxidant and anti-inflammatory (48). In addition, studies had shown that the antioxidant activity of HDL could be significantly affected by the modulation of HDL surface lipids. The surface phospholipid composition of HDL also influenced the anti-inflammatory, anti-apoptotic and anti-infective effects of HDL (49). In conclusion, not only did the content of HDL-associated phospholipids and their ability to neutralize endotoxins contributed to the prevention of sepsis, but their antioxidant, anti-inflammatory, anti-apoptosis, and anti-infective effects were also beneficial for sepsis. Further research is needed to identify the underlying mechanisms behind this relationship.

This study had several strengths, including resistance to confounding factors in traditional epidemiology. We used a variety of MR analysis methods to obtain more reliable data. This study was more efficient and less costly than RCTs. In addition, instrumental variables with larger sample sizes were selected from recent GWAS studies to ensure adequate statistical power. However, it is important to note the limitations of this study. As our study only included European populations, the data do not apply to extrapolations from other non-European populations. Further research on more diverse populations is required.

## Conclusion

Herein, we identified suggestive causal relationship associations between HDL-associated phospholipids levels and the risk of sepsis. These data could aid the development of novel strategies for the diagnosis and treatment of sepsis. However, more research is needed to further explore this question.

## Data availability statement

Publicly available datasets were analyzed in this study. This data can be found here: Summary statistics were publicly available in the GWAS catalog (https://www.ebi.ac.uk).

## Author contributions

LZ: Data curation, Writing – original draft, Writing – review & editing. HT: Methodology, Writing – review & editing. JC: Investigation, Methodology, Writing – review & editing. YiD: Validation, Writing – review & editing. YZ: Validation, Writing – review & editing. HL: Software, Writing – review & editing. YW: Investigation, Writing – review & editing. YP: Investigation, Writing – review & editing. YoD: Conceptualization, Funding acquisition, Project administration, Writing – original draft, Writing – review & editing.
