# Supplementary material for: Causal association of lipoprotein-associated phospholipids on the risk of sepsis: a Mendelian randomization study
Source: Front Endocrinol (Lausanne). 2024 Jan 11;14:1275132. doi: 10.3389/fendo.2023.1275132 (PMC10808779; doi:10.3389/fendo.2023.1275132)
Supplement: Supplementary file 1 [file DataSheet_1.docx]

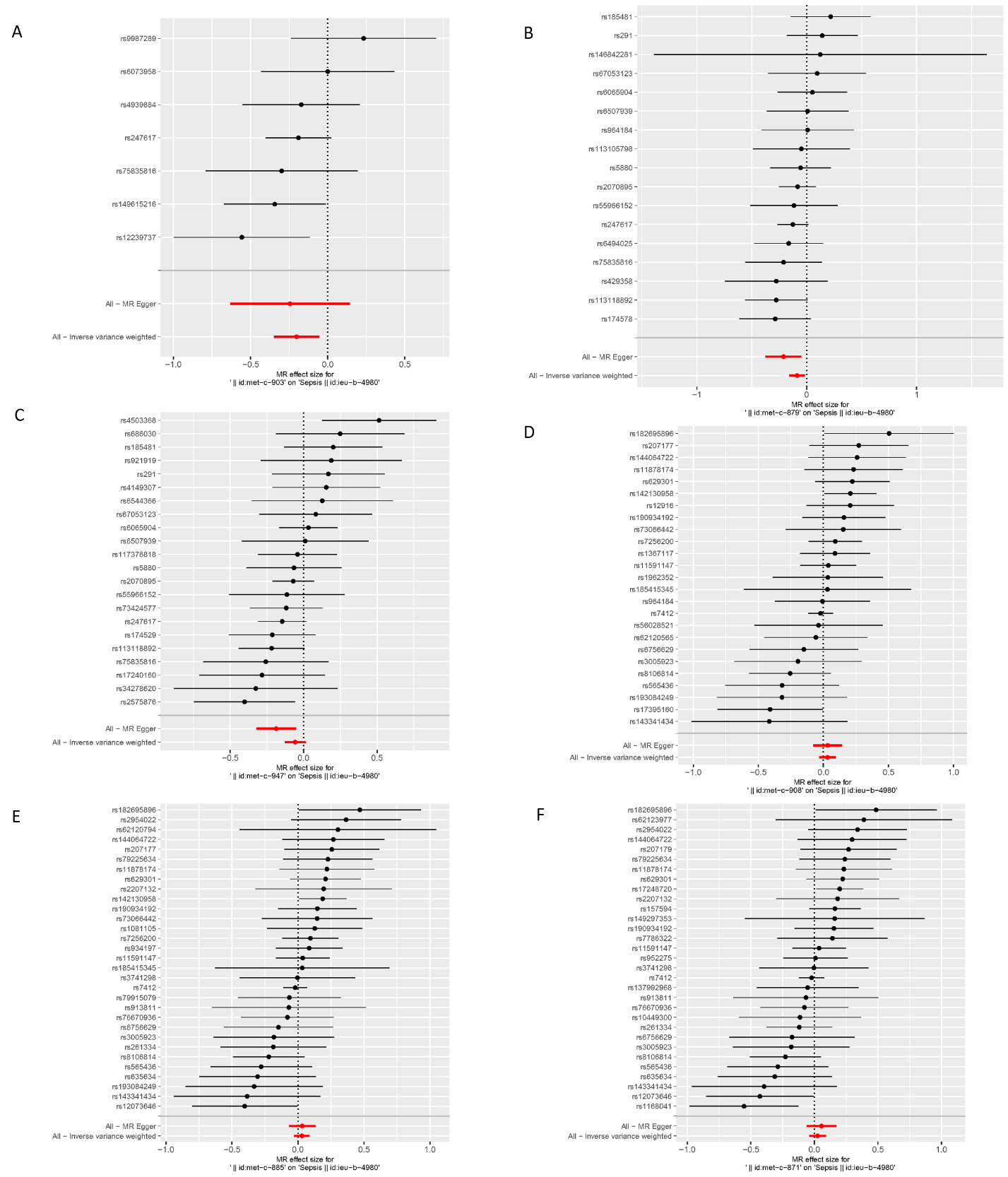


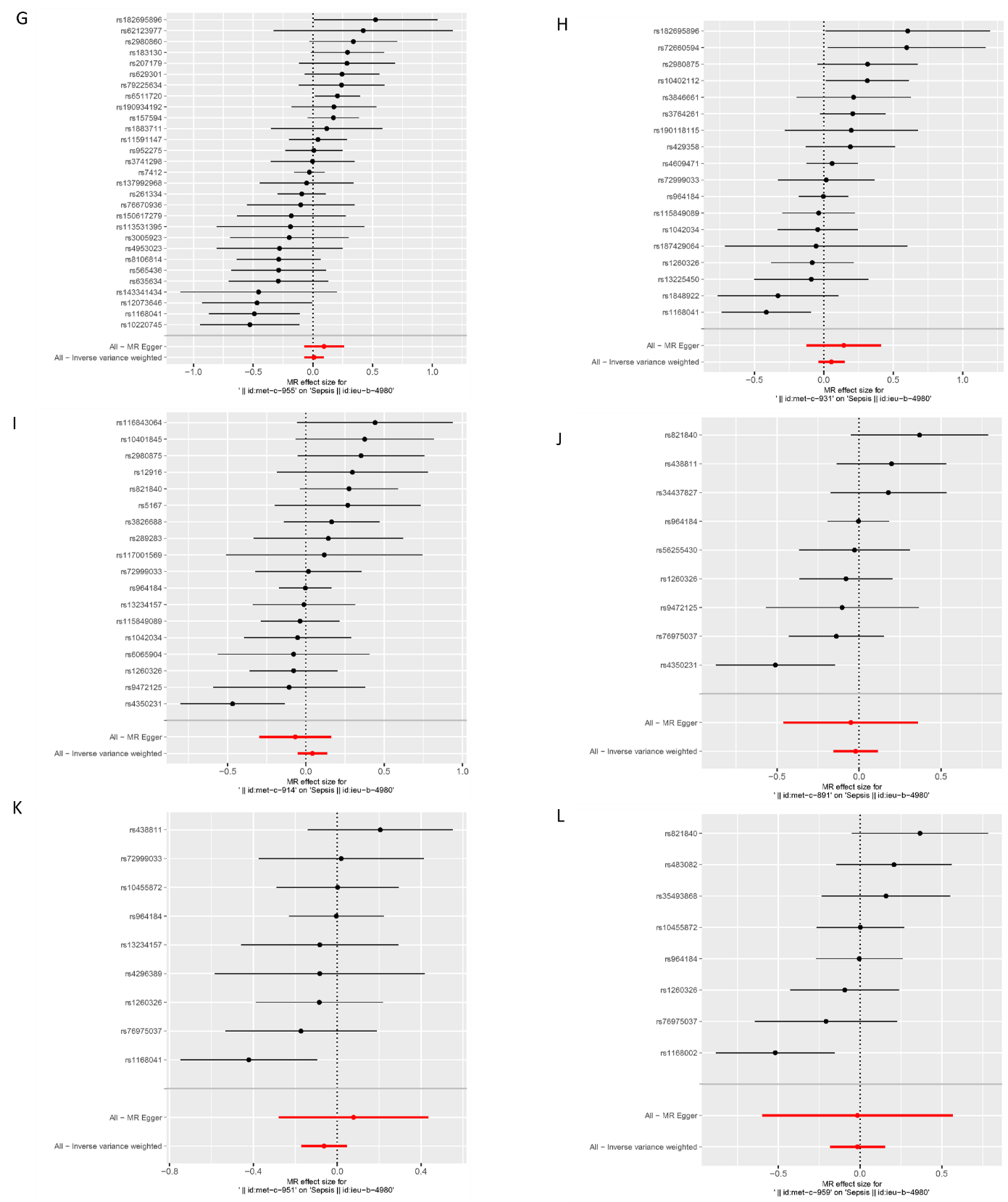


Supplementary Figure S1 Forest plot of the causal relationship between lipoprotein-associated phospholipids and sepsis. A. Phospholipids in medium HDL. B. Phospholipids in large HDL. C. Phospholipids in very large HDL. D. Phospholipids in medium LDL. E. Phospholipids in large LDL. F. Phospholipids in IDL. G. Phospholipids in very small VLDL. H. Phospholipids in small VLDL. I. Phospholipids in medium VLDL. J. Phospholipids in large VLDL. K. Phospholipids in very large VLDL. L. Phospholipids in chylomicrons and largest VLDL particles. The black dots represent the OR value obtained by each method and the solid line represents the 95% CI. MR, Mendelian randomization; OR, odds ratio; CI, confidence interval.


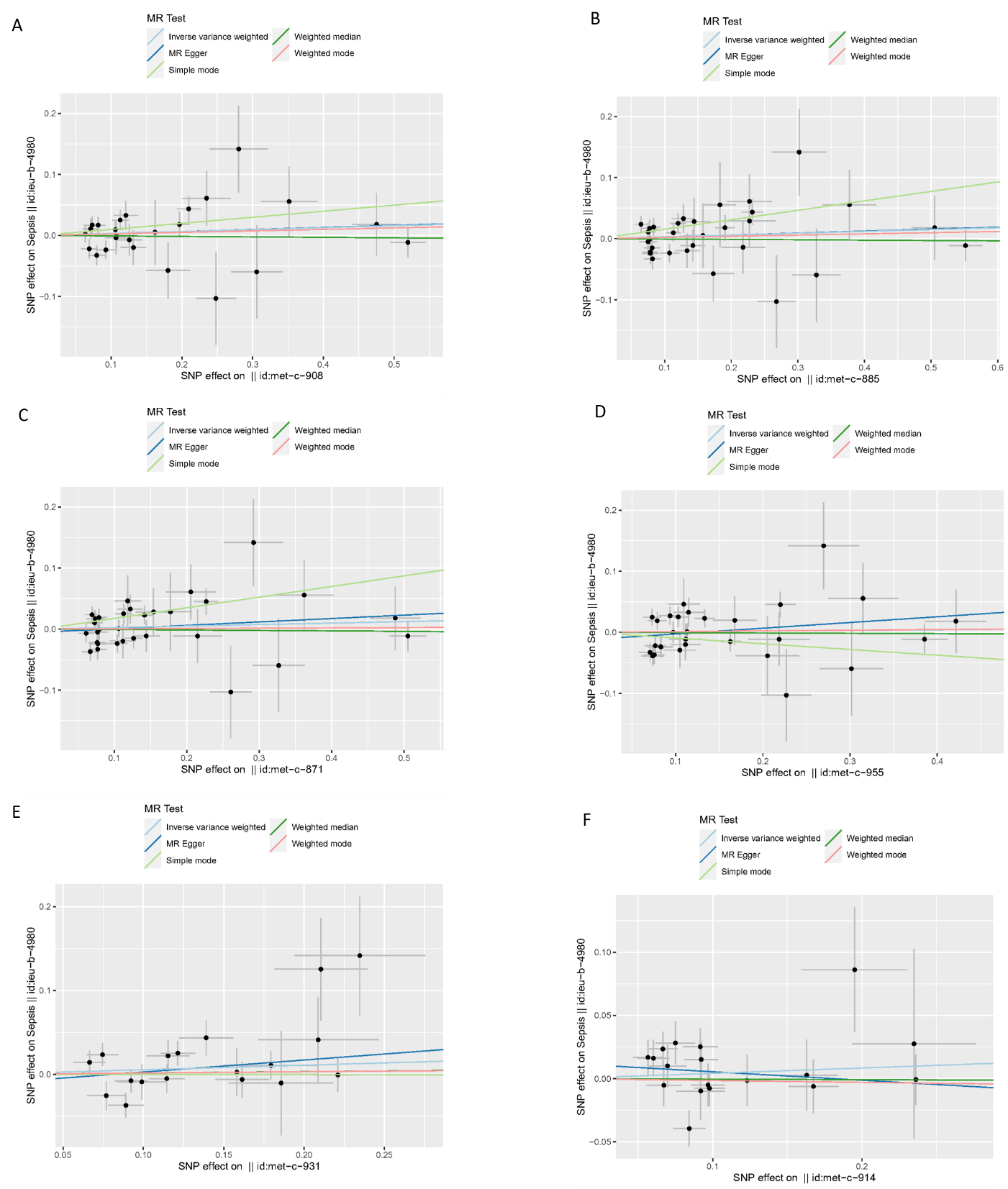


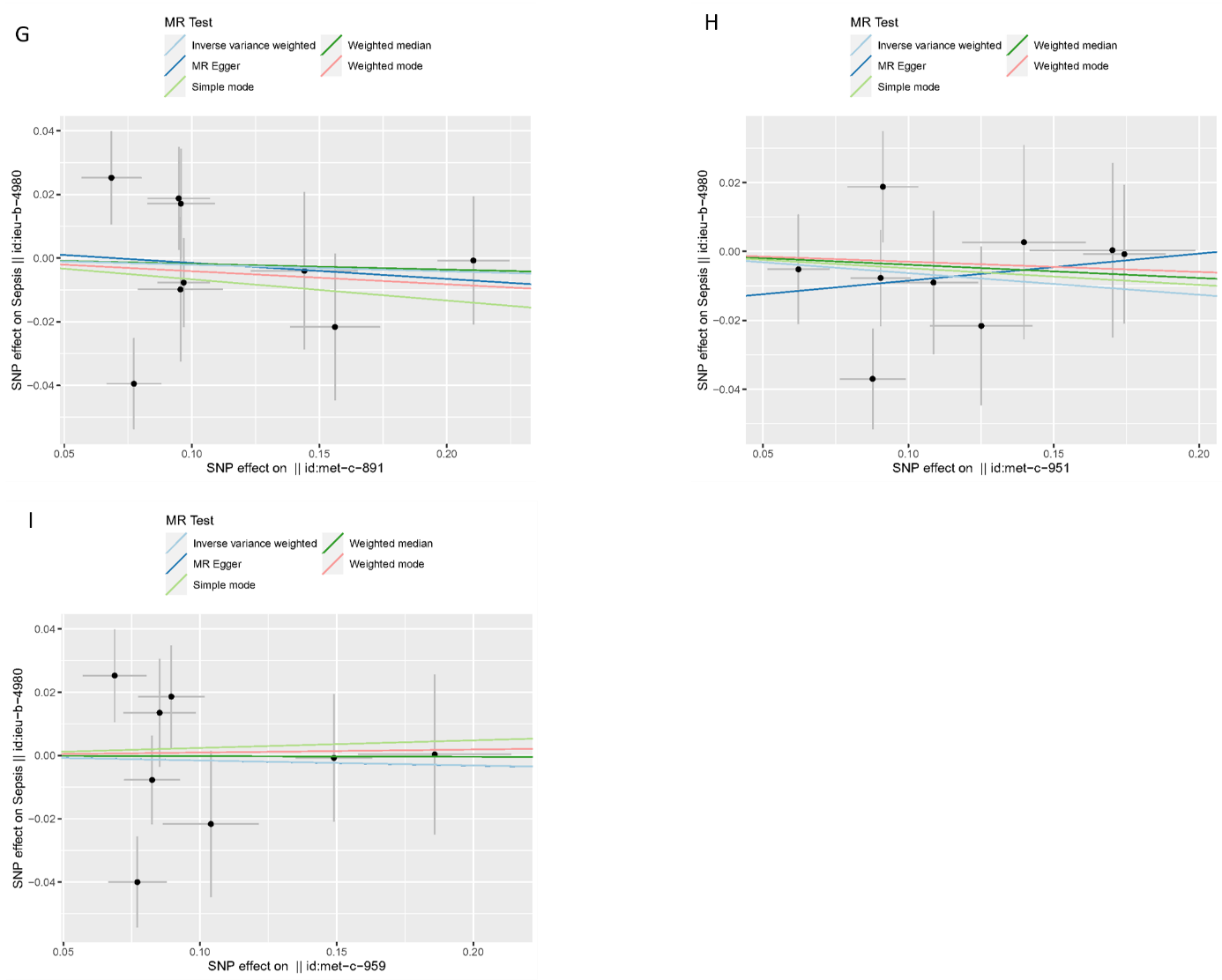


Supplementary Figure S2 Scatter plot of the causal relationship between lipoprotein-associated phospholipids and sepsis. A. Phospholipids in medium LDL. B. Phospholipids in large LDL. C. Phospholipids in IDL. D. Phospholipids in very small VLDL. E. Phospholipids in small VLDL. F. Phospholipids in medium VLDL. G. Phospholipids in large VLDL. H. Phospholipids in very large VLDL. I. Phospholipids in chylomicrons and largest VLDL particles. Analyses were conducted using IVW, weighted median, weighted mode, simple mode and MR Egger methods. The slope of the line indicates the magnitude of the causal relationship. Error bars indicate 95% CI. MR: mendelian randomization; SNP: single nucleotide polymorphism.


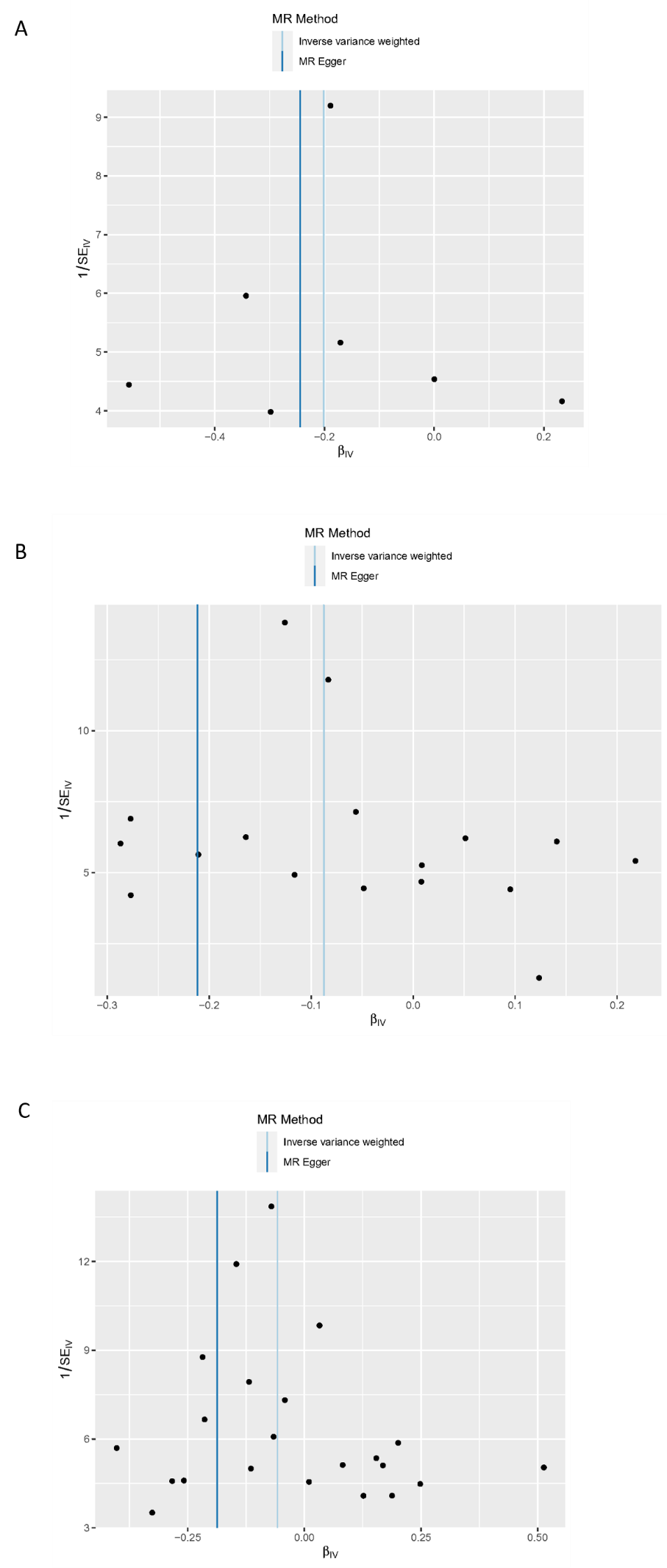


Supplementary Figure S3 Funnel plot for the overall heterogeneity in the effect of HDL-associated phospholipids on sepsis. A. Phospholipids in medium HDL. B. Phospholipids in large HDL. C. Phospholipids in very large HDL.


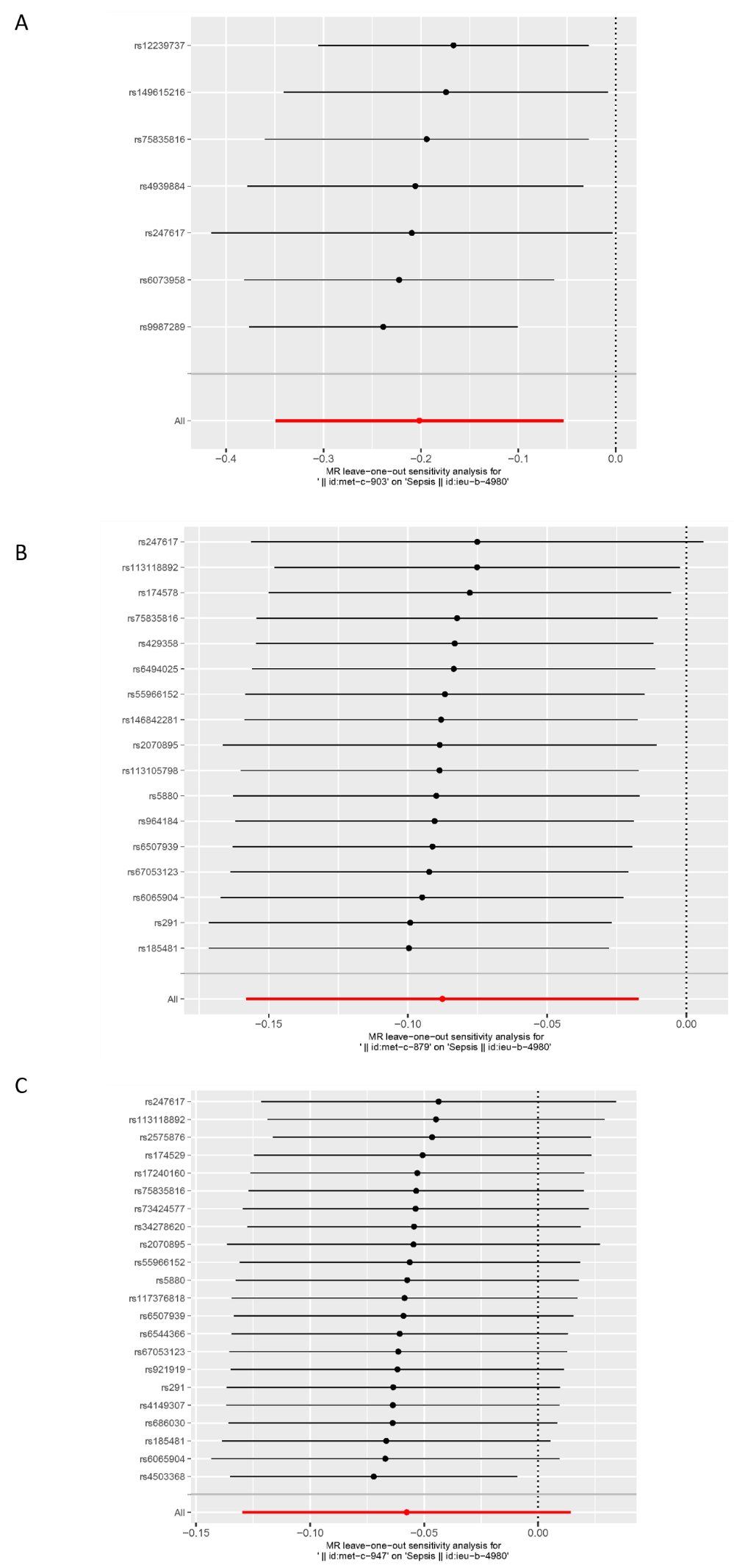


Supplementary Figure S4 Leave-one-out analysis of the effect of HDL-associated phospholipids on sepsis. A. Phospholipids in medium HDL. B. Phospholipids in large HDL. C. Phospholipids in very large HDL.
